# Fluids and Early Vasopressors in the Management of Septic Shock: Do We Have the Right Answers Yet?

**DOI:** 10.2478/jccm-2023-0022

**Published:** 2023-07-31

**Authors:** E. Carlos Sanchez, Michael R. Pinsky, Sharmili Sinha, Rajesh Chandra Mishra, Ahsina Jahan Lopa, Ranajit Chatterjee

**Affiliations:** Department of Critical Care Medicine, King Salman Hospital, Riyadh, Saudi Arabia; Department of Critical Care Medicine, University of Pittsburgh School of Medicine, Pittsburgh, PA, USA; Department of Critical Care Medicine, Apollo Hospitals, Bhubaneswar, India; Department of Critical Care Medicine, Ahmedabad Khyati Multi-speciality Hospitals, Ahmedabad, India Department of Critical Care Medicine, Ahmedabad Shaibya Comprehensive Care Clinic, Ahmedabad, India; ICU and Emergency Department, Shahabuddin Medical College Hospital, Dhaka, Bangladesh; Department of Critical Care Medicine, accident and emergency, Swami Dayanand Hospital Delhi, India

**Keywords:** septic shock, sepsis, vasopressor, fluid, early norepinephrine

## Abstract

Septic shock is a common condition associated with hypotension and organ dysfunction. It is associated with high mortality rates of up to 60% despite the best recommended resuscitation strategies in international guidelines. Patients with septic shock generally have a Mean Arterial Pressure below 65 mmHg and hypotension is the most important determinant of mortality among this group of patients. The extent and duration of hypotension are important. The two initial options that we have are 1) administration of intravenous (IV) fluids and 2) vasopressors, The current recommendation of the Surviving Sepsis Campaign guidelines to administer 30 ml/kg fluid cannot be applied to all patients. Complications of fluid over-resuscitation further delay organ recovery, prolong ICU and hospital length of stay, and increase mortality. The only reason for administering intravenous fluids in a patient with circulatory shock is to increase the mean systemic filling pressure in a patient who is volume-responsive, such that cardiac output also increases. The use of vasopressors seems to be a more appropriate strategy, the very early administration of vasopressors, preferably during the first hour after diagnosis of septic shock, may have a multimodal action and potential advantages, leading to lower morbidity and mortality in the management of septic patients. Vasopressor therapy should be initiated as soon as possible in patients with septic shock.

## Introduction

Septic shock is a common condition associated with hypotension and organ dysfunction [1]. It is associated with high mortality rates of up to 60% in many places around the world, despite the best recommended resuscitation strategies in international guidelines [2]. Septic shock is characterized by systemic vasodilatation and vascular leakage arising from systemic inflammation induced by serious infection [3]. Patients with septic shock generally have a Mean Arterial Pressure (MAP) below 65 mmHg, but this is not a rigid criterion, since many patients could maintain adequate perfusion and function of their organs with even lower values [4]. Another group of patients might need higher MAP values, because they are chronically hypertensive and the renal blood flow autoregulation curve is shifted to the right and they would need values of around 80 mmHg [5], or in patients with high central venous pressure, with intra-abdominal hypertension/abdominal compartment syndrome [6], increased intracranial pressure, and a few other pathological states. Low diastolic arterial pressure (DAP) (< 60 mmHg) has been advocated as an indicator of vasoplegia [7]. Irrespective of the cause, long standing hypotension is associated with worse outcomes. However, its use in initiating vasopressor therapy has not been studied.

## Understanding the septic shock

Septic shock is a type of distributive shock, more particularly vasoplegic, produced by the activation of inducible NO synthetase, which leads to vasodilation and hyperpolarization of vascular smooth muscle cells, making them less responsive to alpha-adrenergic stimulation. This combination usually causes systemic hypotension, with consequent impairment of organ perfusion [8]. Hypotension is the most important determinant of mortality among patients with septic shock. The extent and duration of hypotension are important. A shorter duration of hypotension (< 15 minutes) is often well tolerated; however, prolonged hypotension only progressively increases the development of organ dysfunction and the risk of death [4]. With this understanding, we assume that the initial priority in the management of patients with septic shock is to restore hemodynamics and initially macrohemodynamics, perhaps the first hemodynamic variable to be targeted during the immediate stage of resuscitation is MAP. However, it is important to understand that several physiological factors are involved in the control of MAP.

According to Ohm's law, the pressure drops across a circuit as a function of flow and resistance; in general, we do not measure or take into account the back pressure on organ blood flow. Under normal conditions, the flow to the periphery stops as its small arteries and arteriole tone exceeds the intralumenal pressure. The critical closing pressure (Pcc) is different across vascular beds, but as the lumped vascular parameter in healthy individuals is approximately 40 mmHg. This is important because MAP-Pcc represents the actual organ input pressure. At very low-pressure states, such as sepsis, Pcc is often the same or slightly higher than the tissue or venous pressure in those specific beds. Importantly, organ perfusion pressure may be independent of MAP if the Pcc varies widely, as is the case in sepsis. Regrettably, it is very difficult to measure Pcc routinely at the bedside during resuscitation. However, back pressure is rarely central venous pressure (CVP), nor does increasing CVP artifactually, as occurs with the institution of positive pressure ventilation, cause blood flow to decrease for a constant MAP.

Nevertheless, measures of systemic pressure are useful for differentiating the causes of hypotension. Under most conditions, hypotension is associated with decreased cardiac output. However, in fluid resuscitated septic patients with vasoplegia, cardiac output is often elevated even though arterial pressure is decreased.

The physician must know what determinant we should act on to improve MAP in patients with septic shock, and the two initial options that we have are 1) administration of intravenous (IV) fluids and 2) vasopressors [1]. Up to this point, there is nothing new with the approach to these patients, but perhaps the way we had been doing it was not the best, and evidence-based medicine is teaching us.

## Important aspects of fluid management during septic shock

For many years, the administration of IV fluids has been used to improve MAP in hypotensive patients. In 2001, Dr. Rivers proposed a septic shock management protocol to administer 30 ml/kg of crystalloid solution to achieve initial hemodynamic stabilization as part of early goal-directed therapy [9]. However, between 2014 and 2017, some studies such as ProMISe [10], ARISE [11], ProCESS [12] and PRISM investigators [13] showed that this early goal-directed therapy using such an amount of fluid was not superior to more conservative management. Adequate and appropriate fluid for resuscitation in septic shock has been a controversial topic for years. The current recommendation of the Surviving Sepsis Campaign (SSC) guidelines to administer 30 ml/kg fluid (although this is designated as a “weak” recommendation based on low-quality evidence) cannot be applied to all patients, especially those with comorbid conditions, such as cardiovascular dysfunction or chronic kidney disease. Although this subgroup of patients may benefit from fluid boluses, the quantity of fluid bolus needs to be individualized [14,15]. Beyond this, not all patients arrive at the same stage or under the same conditions; some of them have already been managed previously and could have fluid overload at the time we receive them; in these conditions, the administration of IV fluids is not an appropriate strategy [14,16]. Complications of fluid over-resuscitation further delay organ recovery, prolong ICU and hospital length of stay, and increase mortality [14,17,18,19,20]. It is a fact that preexisting cardiovascular status remains unknown till echocardiography is performed in the absence of exact medical history obtained. Ultrasound machines are not readily available in many neither emergency departments, nor ICUs, or staff is untrained in their use [20,21]. Therefore, stepwise fluid resuscitation with guarded clinical monitoring and starting vasopressors after the administration of an intravenous fluid bolus will be a useful and practical modality to address hypotension in patients presenting with septic shock in the ER and ICU as well [14,22,23].

The devices and maneuvers to monitor fluid responsiveness remain elusive even after decades of research. Various monitoring techniques have been tested and applied in clinical settings to assess and monitor the accurate or near-accurate amount of fluid to be administered [24]. With the advent of new, less invasive, real-time, replicable monitoring techniques, a better understanding of the physiology of critically ill patients, studies in more homogeneous populations, and the use of ultrasonography, it has been shown that dynamic hemodynamic indices are more useful in predicting fluid responsiveness than static indices such as heart rate and blood pressure, among others [24,25,26]. As a result of these advances, it has been sought to know more and more exactly which patient could really benefit from the administration of fluids, with the least amount possible to obtain the answer. This fact contrasts significantly with the recommendations of the best-known guidelines for the management of patients with septic shock, such as the SSC guidelines, which continue suggesting the administration of at least 30 ml/kg of crystalloids [27]. This raises the question of whether this recommendation works for all patients. Does one size fit everyone? Although the protocols guide us on the steps that we should follow to manage our patients, they need to be customized. Sepsis has multiple phenotypes [15], both in patients with and without shock; therefore, the trend is to seek therapies that are as personalized as possible according to the resources we have [28,29].

Occasionally, hypovolemia and always reduced peripheral vascular tone are the two predominant pathophysiological mechanisms responsible for septic shock [8]. With this background, when a patient comes with sepsis and septic shock to the ER/ICU, along with fluid resuscitation, vasopressors are generally started. Although the literature and SSC guidelines now recommend starting vasopressors early, it is not clear how early vasopressors should be started.

In patients with septic shock and dehydration, there is no doubt that fluid deficits must be replenished. For example, patients who may have been vomiting, diarrhea, advanced capillary leak, large burns, acute kidney injury in the polyuric phase, diabetic ketoacidosis, hyperosmolar states, among others, will all demand some level of fluid resuscitation. The situation becomes more complex when a patient appears normovolemic. The idea is to use a maneuver that allows us to know if he could benefit from the administration of fluids as soon as possible, since this hemodynamic property of responding to the administration of fluids decreases with the passage of time, even when patients seem to respond to fluids when performing different maneuvers [16]. Many times, the condition is such that we could initially carry out an initial fluid load of 300–500 ml to evaluate the possible response without further monitoring, but after it, the administration should be better valued to avoid unnecessary and often harmful administration of fluids [14,29]. The assessment and interpretation of the possible response to IV fluids in patients with increased intra-abdominal pressure or ARDS may be more complex and include more specific maneuvers [31].

In the CLOVERS trial, all-cause mortality at 90 days was similar between the fluid restrictive and fluid liberal groups. However, the patients were randomized to either group after they received 1–3 litres of fluid. The incidence of serious events was similar in both groups [32]. Regrettably, none of the subjects were assessed for their degree of volume responsiveness prior to randomization; therefore, many non-volume-responsive patients in the liberal fluid group received a large amount of fluids, and many volume-responsive patients in the restricted volume group did not.

The only reason for administering IV fluids in a patient with circulatory shock is to increase the mean systemic filling pressure in a patient who is volume-responsive, such that cardiac output also increases. However, the mean systemic pressure increases in all patients administered fluids. Guerin et al. showed that fluid increases the mean systemic pressure in all patients regardless of whether or not cardiac output also increases; they also found that the venous return pressure gradient increased only in preload responsiveness patients [33,34]. On the other hand, Monge García et al. found that in those patients who increased their cardiac output before the administration of fluids, the calculated SVR decreased after volume administration, possibly due to decreased sympathetic tone and potentially hemodilution decreasing laminar flow resistance [35]. Therefore, the administration of fluids could have a potentially favorable effect on the increase in MAP only if the increase in cardiac output exceeds the decrease in vasomotor tone. In septic shock, many patients present with high cardiac output due to prior fluid resuscitation[36] (or even without fluid resuscitation), thus using this argument to administer more fluids could be questionable.

## Use of vasopressors during reanimation of septic shock

At the outset of resuscitation from sepsis-induced hypotensive shock, the use of vasopressors seems to be a more appropriate strategy since patients with septic shock have an alteration in vascular tone and, in theory, by improving the vascular tone with the use of either catecholamine or non-catecholamine vasopressors, it could be restored. At least macrohemodynamics, it has been shown in multiple studies, including CENSER [37], that the earlier the vasopressor is administered, the better hemodynamic results and in terms of survival could be obtained, including a lower incidence of cardiogenic pulmonary edema and cardiac arrhythmias.

The very early administration of vasopressors, preferably during the first hour after diagnosis of septic shock, may have a multimodal action. It can cause an increase in preload and cardiac output, reduce preload dependency, and help to improve parameters such as central venous pressure, end-diastolic area of the left ventricle, global end diastolic volume, and E wave, all of them preload parameters [38]. This is probably due to the redistribution of blood volume from the splanchnic circulation to the inferior vena cava, which increases preload, as would be done by intravenous fluids, shifting blood volume from a non-stressed to a stressed volume state [39,40]. This appears to help patients with septic shock, in whom the non-stressed volume is abnormally increased and has the risk of being overloaded by further fluid boluses [41]. Apart from the vasopressor effect of norepinephrine, which increases afterload of the left ventricle, unlike phenylephrine, norepinephrine has beta-adrenergic inotropic effects, thus improving left ventricular systolic function indices such as ejection fraction or Velocity Time Integral (VTI). This could be achieved by two potentially involved mechanisms: 1) action on myocardial β1-receptors not yet downregulated in the early phase and 2) rise in coronary perfusion pressure through an increase in DAP [42].

On the other hand, the early administration of vasopressor takes the MAP to a certain value that is variable according to each patient, but that could recruit collapsed capillaries and thus not only improve hemodynamics but also favor organ flow and, therefore, tissue oxygen saturation of different organs [43]. All of these potential benefits, hence, minimize the need for excessive fluid resuscitation, which significantly reduces the possibility of fluid overload and increases the chance of survival. As shown in the SOAP study, fluid overload is one of the greatest determinants of the 28-day mortality in these patients [17]. Ospina et al. demonstrated that the delayed use of vasopressors (D-VPs) (i.e., norepinephrine) could lead to the administration of fluid volumes as high as 50 ml/kg in some patients during the first 8 h if MAP and blood flow are to be restored with fluids alone. These D-VPs patients had a lower survival rate than those who received very early vasopressors (VE-VPs). They also showed the VE-VP patients received significantly less resuscitation fluids at vasopressor initiation (0[0–510] vs. 1500[650–2300] mL, *p* < 0.001) and during the first 8 h of resuscitation (1100[500–1900] vs. 2600[1600–3800] mL, *p* < 0.001), although the development of acute renal failure and/or the need for renal replacement therapy requirements were not dissimilar. However, VE-VPs were related to a significantly lower net fluid balance 8 and 24 h after VPs. VE-VPs were also associated with a significant reduction in the risk of death compared with D-VPs (HR 0.31, CI95% 0.17–0.57; *p* < 0.001) at day 28. Such an association was also maintained after including patients receiving vasopressors for <6 h [44].

In support of these recent findings, the recent SSG hour-1 bundle now recommends applying vasopressors within the first hour when fluid administration is insufficient to achieve hemodynamic resuscitation goals [45]. In a recent publication thirty-four experts formulated recommendations to start vasopressors early before full completion of fluid resuscitation [46]. Such a practice is still far from implementation, as most intensivists start vasopressors only after complete fluid resuscitation or after establishing that preload independence has been achieved. The mechanisms by which early vasopressor therapy might be beneficial in early septic shock include earlier correction of systemic hypotension and prevention of prolonged organ hypoperfusion [47]. Retrospective evidence has shown that both the degree and duration of hypotension in the initial phase of septic shock are key determinants of patients’ outcome [4]. The time to achieve a MAP target of 65 mmHg was shorter when NE was initiated within the first 6 h of resuscitation compared to a more delayed initiation, as evident from a retrospective study [48].

There are important reasons for starting norepinephrine as soon as possible in hypotensive patients with septic shock.

– Septic shock showed that the time to achieve MAP >65 mmHg was significantly shorter when NE was initiated together with fluid infusion than when NE was initiated only if 30 mL/kg crystalloids failed to achieve the target MAP [4,49,51,59].
– Early NE infusion could augment cardiac output through several mechanisms as previously mentioned [52,53,54,55,59].
– Early NE administration may potentially recruit microvessels and improve microcirculation in cases of severe hypotension through an increase in organ perfusion pressure and decreased sympathetic tone [56,59].
– Early NE administration should prevent fluid overload [17,48,57,58,59].
– Thus, early NE administration should improve outcomes in patients with hypotensive septic shock [48,59].

Permpikul et al. (CENSER trial) found that in the early use of norepinephrine in septic shock, the median time from emergency room arrival to norepinephrine administration was significantly shorter in the early norepinephrine group (93 vs. 192 min; *p* < 0.001). Shock control rate by 6 hours was significantly higher in the early norepinephrine group (118/155 [76.1%] vs. 75/155 [48.4%]; *P* < 0.001). No differences in mortality between the groups were found at 28 days. A lower incidence of cardiogenic pulmonary edema and new-onset arrhythmia were found in the early norepinephrine group [32]. Clearly, prolonged hypotension is associated with worse outcomes [4]. Historically, early use of NE in sepsis resuscitation without adequate fluid resuscitation to support circulating blood volume may cause pathological microvascular vasoconstriction. Elbouhy et al. started NE at a dose of 5 μg/min along with fluid resuscitation in the ER in patients with septic shock. The time to achieve a target MAP >65 mmHg was 2 h compared to 3 h in the late vasopressor group, where NE was administered after fluid resuscitation of 30 ml/kg [60]. In light of the current evidence, clinical data favor the early use of vasopressors in patients with septic shock. However, there is no consensus on the optimal starting time. We favor starting NE if after the initial IV fluid bolus of 5–10 ml/kg the patient remains hypotensive.

An International multicenter retrospective analysis of 2,849 patients with septic shock suggested that starting NE between 1 and 6 h after starting fluid infusion could result in better outcomes [59], these results contrast with those obtained in a recent CRT single center where the best outcomes were obtained when starting NE very early 25 min (20–30) min after diagnosing the septic shock state compared to the late group 120 min (120–180) [50].

A simple way to identify patients who urgently need NE is to assess the peripheral measures of arterial tone. The DAP is a function of sympathetic tone and heart rate. If DAP is <60 mmHg, it is mainly due to a depressed vascular tone, especially in the case of tachycardia. Such low diastolic pressure has been advocated with vasoplegia [59,62]. Holder et al. found that DAP <52 mmHg independently predicted early progression to septic shock. The lower the DAP, the greater the vasoplegia, which is directly related with mortality [63]. Ospina-Tascon et al. described the Diastolic Shock Index (DSI), which is the ratio of heart rate over DAP (DSI=HR/DBP). A DSI > 2.2 was associated with higher mortality in septic shock; the higher the value obtained, the greater the mortality. Thus, measuring a low DAP in this context should prompt urgent initiation of NE, even in the absence of central venous access [64].

However, it must be borne in mind that there are situations in which DBP may be low without this being associated with infection-induced vasoplegia, among them we have: long bed rest, dehydration, alcohol intake, hormonal deficiencies and other endocrine dysfunctions such as hypothyroid or excess blood loss in menstruation, pregnancy, allergic reactions, anemia (deficiency of B vitamins, iron, folate), aortic aneurism, aortic valve regurgitation, medications (beta and alpha-blockers, erectile dysfunction drugs, tricyclic antidepressants, Parkinson's disease drugs, diuretics) among some others, and even in some patients with septic shock these conditions can also be present and they have to make us think that perhaps the blood pressure target is lower than usual.

## The down side of early vasopressor therapy

The SSC guidelines recommend starting a vasopressor after fluid administration if hypotension has not been corrected or even during fluid infusion [27], but we know that arterial hypotension is a direct determinant of mortality and that in terms of time arterial hypotension can be corrected faster using vasopressor, and understanding that these patients do not have fluid losses, then should we really administer fluids first?

The disadvantage of starting fluids and vasopressors at the beginning of resuscitation to achieve a target MAP of 65 mmHg early may “hide” an underlying fluid deficit, promoting further tissue hypoperfusion despite increasing the MAP. Such a strategy of early NE may hide coexisting low cardiac output (CO) values. The intravascular volume needs to be adequate to sustain venous return for increased vasomotor tone to increase MAP without inducing tissue hypoperfusion. However, excessive fluids during and after the resuscitative phase are well-known harmful situation [14,16].

## The early combination of fluids and norepinephrine

**Fig. 1.**
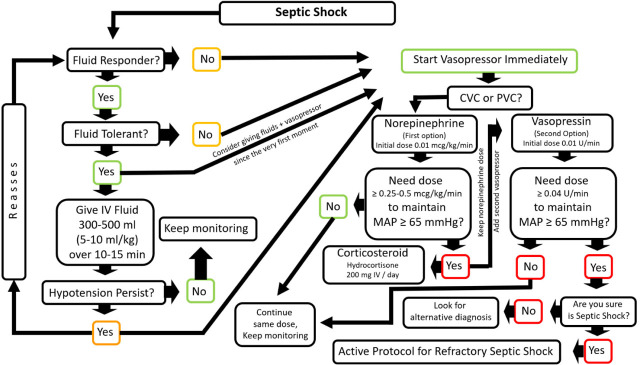
Rational approach and management of septic shock with intravenous fluids and early vasopressors based on current evidence. IV: Intravenous, CVC: Central Venous Catheter, PVC: Peripheral Venous Catheter.

The early combination of fluids and norepinephrine, most likely during the first hour, has potential advantages in the management of patients with septic shock of rapidly increasing mean systemic filling pressure more than fluids alone would, thereby achieving better CO in volume-responsive patients in the setting of a higher MAP; corrects hypotension and perfusion better than fluid or vasopressor alone; decreases the possibility of fluid overload; may improve tissue oxygenation, although this has not been substantiated; and leads to lower morbidity and mortality in the management of septic patients [44,61] (Figure 1).

## Vasopressor options when norepinephrine is not available

The SSC guidelines 2021 mention this regarding norepinephrine use in settings where it is not available. “Epinephrine or dopamine can be used as an alternative, but we encourage efforts to improve the availability of norepinephrine. Special attention should be given to patients at risk for arrhythmias when using dopamine and epinephrine. For adults with septic shock on norepinephrine with inadequate MAP levels, we suggest adding vasopressin instead of escalating the dose of norepinephrine *(Weak recommendation, moderate-quality evidence)*. Vasopressin is usually started when the dose of norepinephrine is in the range of 0.25–0.5 μg/kg/min. For adults with septic shock and inadequate MAP levels despite norepinephrine and vasopressin, we suggest adding epinephrine (*Weak recommendation, low-quality evidence)”* [27].

However, when norepinephrine is available, the use of epinephrine or dopamine in patients with septic shock is reserved for those patients who, in addition to low blood pressure, have low cardiac output not associated with hypovolemia, but altered cardiac performance.

The effect of early vasopressin initiation on clinical outcomes in patients with septic shock remains uncertain. A meta-analysis of early initiation of vasopressin in patients with septic shock showed that its use was not associated with decreased short-term mortality, new-onset arrhythmias, shorter ICU length of stay, or length of hospitalization, but it did reduce the use of RRT [65]. Gordon et al. (VANISH trial) compared the effect of early vasopressin vs. norepinephrine on kidney failure in patients with septic shock and found that among adults with septic shock, the early use of vasopressin compared to norepinephrine did not improve the number of kidney failure–free days [66]. However, other researchers, such as Rydz et al., found that early initiation of vasopressin in septic shock may reduce the risk of in-hospital all-cause mortality and/or organ dysfunction [67]. So, is vasopressin for all patients? Nakamura et al. found that vasopressin loading might predict responses to continuous administration in patients with septic shock. A 1 U bolus of vasopressin was administered, followed by continuous administration at 1 U/h. They defined responders as patients with a MAP increase >18 mmHg 1 min after vasopressin loading [68]. These patients had beneficial outcomes with the use of vasopressin.

## Do corticosteroids play any role?

Although there is no conclusive evidence that the routine use of glucocorticoids in patients with septic shock improves survival, a recent meta-analysis showed that it can help shorten the reversal time of septic shock. Hydrocortisone is the one glucocorticoid, that has showed the best results. There is still no consensus regarding the best time for administration, vasopressor dose, administration time, or whether the dose should be personalized [69]. However, the SSC guidelines recommend start hydrocortisone 200 mg/day IV, either as a bolus every 6 hours or as a continuous infusion if the patient with septic shock fails to stabilize after fluid resuscitation and require the use of vasopressors (norepinephrine or epinephrine ≥ 0.25 mcg/kg/min at least 4 hours after initiation) [27]. Currently, there are no recommendations regarding corticosteroid use when starting treatment with another vasopressor.

## Starting vasopressors peripherally?

For adults with septic shock, the SSC suggest starting vasopressors peripherally to restore MAP rather than delaying initiation until central venous access is secured [60,61,62,63,64,65,66,67,68,69,70]. A recent prospective clinical trial of norepinephrine infusion using peripheral intravenous access in over 1000 patients showed no adverse events [71,72,73]. Thus, one needs not wait for a central venous access port to start norepinephrine infusion. Regrettably, despite these data, there is no consensus regarding the moment or dose of vasopressors at which it should be administered by central venous catheter [73].

Many questions regarding the use of early vasopressors remain unanswered. First, it is not clear whether it is better to administer vasopressors first in a hypotensive septic patient followed by fluids, or whether fluids and vasopressors should be administered simultaneously. We prefer to give them simultaneously, but give the fluid in bolus form and then assess its effects 15 min later. Second, it is unclear which vasopressors should be used. Norepinephrine is the primary vasopressor, but vasopressin, dopamine, and angiotensin-2 are potent vasopressors with different vascular bed vasopressor profiles. This leads us to another two fields: the personalized use of vasopressors and broad-spectrum vasopressor therapy as part of the initial management of septic shock [74]. Perhaps all these strategies are appropriate, as long as the fluid therapy is personalized, as something that seems as simple as administering fluids can become a very complex process with catastrophic consequences for the patient when it is not done correctly. It is prudent to determine beforehand the volume responsiveness in a patient with shock [75].

## Conclusions

Based on current evidence, the very early use of norepinephrine and its combination with smaller amounts of IV fluids in fluid-responsive hypotensive septic shock patients seems to be a safe strategy with better management results compared to large-volume fluids before vasopressor initiation. Vasopressor therapy should be initiated as soon as possible in patients with septic shock.

## References

[1] Singer M, Deutschman CS, Seymour CW (2016). The Third International Consensus Definitions for Sepsis and Septic Shock (Sepsis-3). JAMA..

[2] Martin-Loeches I, Guia MC, Vallecoccia MS (2019). Risk factors for mortality in elderly and very elderly critically ill patients with sepsis: a prospective, observational, multicenter cohort study. Ann. Intensive Care..

[3] Jarczak D, Kluge S, Nierhaus A (2021). Sepsis—Pathophysiology and Therapeutic Concepts. Front. Med..

[4] Varpula M, Tallgren M, Saukkonen K, Voipio-Pulkki LM, Pettilä V (2005). Hemodynamic variables related to outcome in septic shock. Intensive Care Med.

[5] Asfar P, Meziani F, Hamel JF, SEPSISPAM Investigators (2014). High versus low blood-pressure target in patients with septic shock. N Engl J Med.

[6] Al-Dorzi HM, Tamim HM, Rishu AH (2012). Intra-abdominal pressure and abdominal perfusion pressure in cirrhotic patients with septic shock. Ann. Intensive Care..

[7] Benchekroune S, Karpati PC, Berton C (2008). Diastolic arterial blood pressure: a reliable early predictor of survival in human septic shock. J Trauma..

[8] Vincent JL, De Backer D (2013). Circulatory shock. N Engl J Med.

[9] Rivers E, Nguyen B, Havstad S, for the Early Goal-Directed Therapy Collaborative Group (2001). Early Goal-Directed Therapy in the Treatment of Severe Sepsis and Septic Shock.

[10] Mouncey P, Osborn T, Power S, for the ProMISe Trial Investigators (2015). Trial of Early, Goal-Directed Resuscitation for Septic Shock. N Engl J Med.

[11] The ARISE Investigators and the ANZICS Clinical Trials Group (2014). Goal-Directed Resuscitation for Patients with Early Septic Shock. N Engl J Med..

[12] Yealy DM, Kellum JA, Huang DT, ProCESS Investigators (2014). A randomized trial of protocol-based care for early septic shock. N Engl J Med.

[13] The PRISM Investigators (2017). Early, Goal-Directed Therapy for Septic Shock — A Patient-Level Meta-Analysis. N Engl J Med..

[14] Malbrain MLNG, Van Regenmortel N, Saugel B (2018). Principles of fluid management and stewardship in septic shock: it is time to consider the four D's and the four phases of fluid therapy. Ann. Intensive Care.

[15] Seymour CW, Kennedy JN, Wang S (2019). Derivation, Validation, and Potential Treatment Implications of Novel Clinical Phenotypes for Sepsis. JAMA.

[16] Silversides JA, Perner A, Malbrain MLNG (2019). Liberal versus restrictive fluid therapy in critically ill patients. Intensive Care Med.

[17] Vincent JL, Sakr Y, Sprung CL, Sepsis Occurrence in Acutely Ill Patients Investigators (2006). Sepsis in European intensive care units: results of the SOAP study. Crit Care Med.

[18] Sakr Y, Rubatto Birri PN, Kotfis K, Nanchal R, Intensive Care Over Nations Investigators (2017). Higher Fluid Balance Increases the Risk of Death From Sepsis: Results From a Large International Audit. Crit Care Med.

[19] van Mourik N, Geerts BF, Binnekade JM (2020). A Higher Fluid Balance in the Days After Septic Shock Reversal Is Associated With Increased Mortality: An Observational Cohort Study. Crit Care Explor.

[20] Hernández G, Kattan E, Ospina-Tascón G, Bakker J, Castro R, ANDROMEDA-SHOCK Study Investigators and the Latin America Intensive Care Network (LIVEN) (2020). Capillary refill time status could identify different clinical phenotypes among septic shock patients fulfilling Sepsis-3 criteria: a post hoc analysis of ANDROMEDA-SHOCK trial. Intensive Care Med.

[21] Slemko JM, Daniels VJ, Bagshaw SM (2021). Critical care ultrasound training: a survey exploring the “education gap” between potential and reality in Canada. Ultrasound J..

[22] Kanji HD, McCallum J, Sirounis D, MacRedmond R, Moss R, Boyd JH (2014). Limited echocardiography-guided therapy in subacute shock is associated with change in management and improved outcomes. J Crit Care.

[23] Ueyama H, Kiyonaka S (2017). Predicting the Need for Fluid Therapy—Does Fluid Responsiveness Work?. J Intensive Care..

[24] Martin ND, Codner P, Greene W, Brasel K, Michetti C, AAST Critical Care Committee (2020). Contemporary hemodynamic monitoring, fluid responsiveness, volume optimization, and endpoints of resuscitation: an AAST critical care committee clinical consensus. Trauma Surg Acute Care Open.

[25] Shrestha GS, Srinivasan S (2018). Role of Point-of-Care Ultrasonography for the Management of Sepsis and Septic Shock. Rev Recent Clin Trials..

[26] Verras C, Ventoulis I, Bezati S, Matsiras D, Parissis J, Polyzogopoulou E (2023). Point of Care Ultrasonography for the Septic Patient in the Emergency Department: A Literature Review. J. Clin. Med..

[27] Evans L, Rhodes A, Alhazzani W (2021). Surviving sepsis campaign: international guidelines for management of sepsis and septic shock 2021. Intensive Care Med.

[28] De Backer D, Cecconi M, Chew MS (2022). A plea for personalization of the hemodynamic management of septic shock. Crit Care..

[29] Zhang Z, Zheng B, Liu N (2020). Individualized fluid administration for critically ill patients with sepsis with an interpretable dynamic treatment regimen model. Sci Rep..

[30] Vincent JL, Cecconi M, De Backer D (2020). The fluid challenge. Crit Care.

[31] Lai C, Monnet X, Teboul JL (2023). Hemodynamic Implications of Prone Positioning in Patients with ARDS. Crit Care..

[32] Shapiro NI, Douglas IS, Brower RG, National Heart, Lung, and Blood Institute Prevention and Early Treatment of Acute Lung Injury Clinical Trials Network (2023). Early Restrictive or Liberal Fluid Management for Sepsis-Induced Hypotension. N Engl J Med.

[33] Persichini R, Lai C, Teboul JL, Adda I, Guérin L, Monnet X (2022). Venous return and mean systemic filling pressure: physiology and clinical applications. Crit Care.

[34] Guérin L, Teboul JL, Persichini R (2015). Effects of passive leg raising and volume expansion on mean systemic pressure and venous return in shock in humans. Crit Care..

[35] Monge García MI, Barrasa González H (2017). Why did arterial pressure not increase after fluid administration?. Med Intensiva.

[36] Vallet B, Pinsky MR, Cecconi M (2013). Resuscitation of patients with septic shock: please “mind the gap”!. Intensive Care Med.

[37] Permpikul C, Tongyoo S, Viarasilpa T, Trainarongsakul T, Chakorn T, Udompanturak S (2019). Early Use of Norepinephrine in Septic Shock Resuscitation (CENSER). A Randomized Trial. Am J Respir Crit Care Med.

[38] Monnet X, Jabot J, Maizel J, Richard C, Teboul JL (2011). Norepinephrine increases cardiac preload and reduces preload dependency assessed by passive leg raising in septic shock patients. Crit Care Med.

[39] Spiegel R (2016). Stressed vs. unstressed volume and its relevance to critical care practitioners. Clin Exp Emerg Med.

[40] Magder S (2016). Volume and its relationship to cardiac output and venous return. Crit Care..

[41] Chalkias A, Laou E, Papagiannakis N (2022). Assessment of Dynamic Changes in Stressed Volume and Venous Return during Hyperdynamic Septic Shock. J. Pers. Med..

[42] Hamzaoui O, Georger JF, Monnet X (2010). Early administration of norepinephrine increases cardiac preload and cardiac output in septic patients with life-threatening hypotension. Crit Care..

[43] Georger JF, Hamzaoui O, Chaari A, Maizel J, Richard C, Teboul JL (2010). Restoring arterial pressure with norepinephrine improves muscle tissue oxygenation assessed by near-infrared spectroscopy in severely hypotensive septic patients. Intensive Care Med.

[44] Ospina-Tascón GA, Hernandez G, Alvarez I (2020). Effects of very early start of norepinephrine in patients with septic shock: a propensity score-based analysis. Crit Care.

[45] Levy MM, Evans LE, Rhodes A (2018). The Surviving Sepsis Campaign Bundle: 2018 update. Intensive Care Med.

[46] Scheeren TWL, Bakker J, De Backer D (2019). Current use of vasopressors in septic shock. Ann. Intensive Care..

[47] Li Y, Li H, Zhang D (2020). Timing of norepinephrine initiation in patients with septic shock: a systematic review and meta-analysis. Crit Care.

[48] Bai X, Yu W, Ji W (2014). Early versus delayed administration of norepinephrine in patients with septic shock. Crit Care.

[49] Colon Hidalgo D, Patel J, Masic D (2020). Delayed vasopressor initiation is associated with increased mortality in patients with septic shock. J Crit Care..

[50] Elbouhy MA, Soliman M, Gaber A (2019). Early use of norepinephrine improves survival in septic shock: earlier than early. Arch Med Res.

[51] Vincent JL, Nielsen ND, Shapiro NI (2018). Mean arterial pressure and mortality in patients with distributive shock: a retrospective analysis of the MIMIC-III database. Ann Intensive Care..

[52] Persichini R, Silva S, Teboul JL (2012). Effects of norepinephrine on mean systemic pressure and venous return in human septic shock. Crit Care Med..

[53] Hamzaoui O, Jozwiak M, Geffriaud T (2018). Norepinephrine exerts an inotropic effect during the early phase of human septic shock. Br J Anaesth..

[54] Monnet X, Jabot J, Maizel J (2011). Norepinephrine increases cardiac preload and reduces preload dependency assessed by passive leg raising in septic shock patients. Crit Care Med..

[55] Hamzaoui O, Jozwiak M, Geffriaud T (2018). Norepinephrine exerts an inotropic effect during the early phase of human septic shock. Br J Anaesth..

[56] Georger JF, Hamzaoui O, Chaari A (2010). Restoring arterial pressure with norepinephrine improves muscle tissue oxygenation assessed by near-infrared spectroscopy in severely hypotensive septic patients. Intensive Care Med..

[57] Macdonald SPJ, Keijzers G, Taylor DM (2018). Restricted fluid resuscitation in suspected sepsis associated hypotension (REFRESH): a pilot randomised controlled trial. Intensive Care Med..

[58] Boyd JH, Forbes J, Nakada T-a (2011). Fluid resuscitation in septic shock: a positive fluid balance and elevated central venous pressure are associated with increased mortality. Crit Care Med..

[59] Shi R, Hamzaoui O, De Vita N, Monnet X, Teboul JL (2020). Vasopressors in septic shock: which, when, and how much?. Ann Transl Med.

[60] Elbouhy MA, Soliman M, Gaber A, Taema KM, Abdel-Aziz A (2019). Early Use of Norepinephrine Improves Survival in Septic Shock: Earlier than Early. Arch Med Res.

[61] Adda I, Lai C, Teboul JL, Guerin L, Gavelli F, Monnet X (2021). Norepinephrine potentiates the efficacy of volume expansion on mean systemic pressure in septic shock. Crit Care.

[62] Hamzaoui O, Shi R (2020). Early norepinephrine use in septic shock. J Thorac Dis.

[63] Holder AL, Gupta N, Lulaj E (2016). Predictors of early progression to severe sepsis or shock among emergency department patients with nonsevere sepsis. Int J Emerg Med.

[64] Ospina-Tascón GA, Teboul JL, Hernandez G (2020). Diastolic shock index and clinical outcomes in patients with septic shock. Ann Intensive Care.

[65] Huang H, Wu C, Shen Q, Xu H, Fang Y, Mao W (2021). The effect of early vasopressin use on patients with septic shock: A systematic review and meta-analysis. Am J Emerg Med.

[66] Gordon AC, Mason AJ, Thirunavukkarasu N, VANISH Investigators (2016). Effect of Early Vasopressin vs Norepinephrine on Kidney Failure in Patients With Septic Shock: The VANISH Randomized Clinical Trial. JAMA.

[67] Rydz AC, Elefritz JL, Conroy M (2022). Early initiation of vasopressin reduces organ failure and mortality in septic shock. Shock.

[68] Nakamura K, Nakano H, Naraba H (2021). Vasopressin Loading for Refractory Septic Shock: A Preliminary Analysis of a Case Series. Front Med.

[69] Gibbison B, López-López JA, Higgins JP (2017). Corticosteroids in septic shock: a systematic review and network meta-analysis. Crit Care.

[70] Legrand M, Zarbock A (2022). Ten tips to optimize vasopressors use in the critically ill patient with hypotension. Intensive Care Med.

[71] Cardenas-Garcia J, Schaub KF, Belchikov YG, Narasimhan M, Koenig SJ, Mayo PH (2015). Safety of peripheral intravenous administration of vasoactive medication. J Hosp Med.

[72] Maksim K, Abhinav A, Zubair H (2018). Safety of peripheral intravenous administration of vasoactive medication: a retrospective one year follow up. Chest..

[73] Liu Liwei, Luo Lan, Li Lu, Jin Mu (2020). Safety of high-concentration norepinephrine for peripheral intravenous use. Comment on Br J Anaesth.

[74] Chawla LS, Ostermann M, Forni L (2019). Broad spectrum vasopressors: a new approach to the initial management of septic shock?. Crit Care.

[75] Monnet X, Lai C, Teboul JL (2023). How I personalize fluid therapy in septic shock?. Crit Care.

